# Identification of lung adenocarcinoma specific dysregulated genes with diagnostic and prognostic value across 27 TCGA cancer types

**DOI:** 10.18632/oncotarget.19823

**Published:** 2017-08-02

**Authors:** Jun Shang, Qian Song, Zuyi Yang, Dongyao Li, Wenjie Chen, Lei Luo, Yongkun Wang, Jingcheng Yang, Shikang Li

**Affiliations:** ^1^ Department of Thoracic and Cardiovascular Surgery, The First Affiliated Hospital of Guangxi Medical University, Nanning 530021, P. R. China; ^2^ Department of Hematology, The First Affiliated Hospital of Soochow University, Suzhou 215006, P. R. China

**Keywords:** RNA-seq, diagnosis, prognosis, lung adenocarcinoma

## Abstract

As the most common histologic subtype of lung cancer, lung adenocarcinoma (LUAD) contributes to a majority of cancer-related deaths worldwide annually. In order to find specific biomarkers of LUAD that are able to distinguish LUAD from other types of cancer so as to improve the early diagnostic and prognostic power in LUAD, we analyzed 10098 tumor tissue samples across 27 TCGA cancer types and identified 112 specific expressed genes in LUAD. Meantime, 8240 LUAD dysregulated genes in tumor and normal samples were identified. Combining with the results of specific expressed genes and dysregulated genes in LUAD, we found there were 70 specific dysregulated genes in LUAD (LUAD-SDGs). Then ROC curve revealed six LUAD-SDGs that may be of strong diagnostic value to predict the existence of cancer (area under curve[AUC] > 95%). Kaplan-Meier survival analysis was performed to identify 6 LUAD-SDGs associated with patients’ prognosis (P-values < 0.001). Multivariate Cox proportional hazards regression was employed to demonstrate that the six LUAD-SDGs were independent prognostic factors. Then, we used the six overall survival (OS)-related LUAD-SDGs constructing a six-gene signature. Multivariate Cox regression analysis suggested that the six-gene signature was an independent prognostic factor of other clinical variables (hazard ratio [HR] = 1.5098, 95%CI = 1.2996-1.7538, P < 0.0001). Based on our findings, we first presented the LUAD-SDGs for LUAD diagnosis and prognosis. Our results may provide efficient biomarkers to clinical diagnostic and prognostic evaluation in LUAD.

## INTRODUCTION

Lung cancer is one of the most frequently diagnosed cancers and contributes to the majority of cancer-related deaths. According to the latest statistics, there were about 1.8 million new lung cancer cases and 1.5 million people who died of lung cancer in the future [1]. As a subtype of lung cancer, lung adenocarcinoma (LUAD) is continuously growing in the proportion of lung cancer, which is presently the top diagnosed histological type in adult men and women [2]. Smoking is the leading cause of lung cancer, but LUAD is the histological type showing weakness associated with smoking, which occurs mainly to non-smokers and females [3, 4]. Oncogene aberrations are now the most popular study factors that contribute to the carcinogenesis of LUAD [5, 6]. Although there are lots of contributions to individualized therapy of LUAD, patients with advanced-stage tumors have an overall survival of less than 2 years, which is mainly because they have lost the chance of surgery with a limiting selection of chemotherapy [7]. Besides, the molecular biomarkers that can effectively predict the outcome of LUAD patients have not been fully elucidated. Therefore it is of significant importance to figure out new LUAD special biomarkers for the diagnosis and prognosis of LUAD and to improve therapeutic effects.

In the genomics era, there emerge a lot of high throughput sequencing technologies and databases. They have contributed to the development of diagnostic and prognostic signatures of cancer. RNA-seq is a recently developed approach to deep-sequencing. It can identify unmapped genes, unrecognized non-coding RNAs and splice variants [8]. *Iris H. Wei* et al used RNA-seq data from international Cancer Genome Consortium and The Cancer Genome Atlas (TCGA) datasets conducting cancer-specific signatures. These signatures had high sensitivity and specificity and were able to identify metastatic cancer of unknown primary, including tumors originating from lung [9]. Besides, by using TCGA lung adenocarcinoma datasets, *Anlin Feng* et al found LUAD patients with high HMGB1 expression had a poor overall survival [10]. These indicate that these newly developed genome sequencing databases and methods can develop clinical biomarkers for LUAD. And there are some signatures which have been investigated to involve in the pathogenesis of lung cancer and have clinical associations with LUAD. Tony *et al* used a loss-of-function model to identify MALAT1 which was an active regulator of LUAD metastasis [11]. Ming *et al*. showed that decreased BANCER expression was associated with poor prognosis of lung cancer patients [12]. Gao *et al* found overexpression of serum miR-155 could be a diagnostic marker for the early detection of LUAD [13]. With the help of the next-generation sequencing biomarkers, we were able to detect the LUAD patients and predict the outcome of these patients in an early stage.

In this paper, in order to identify LUAD-SDGs, we analyzed large scale RNA-seq transcriptomes of 10098 tumor samples across 27 cancer types containing LUAD from TCGA. Meanwhile, dysregulated genes in LUAD were identified. Combining with the diagnostic and survival analysis, we found robust diagnostic and prognostic LUAD-SDGs. By utilizing the prognostic LUAD-SDGs, we conducted a six-gene signature that could effectively predict patients’ survival.

## RESULTS

### Identification of specific dysregulated genes of lung adenocarcinoma (LUAD-SDGs)

**Figure 1:**
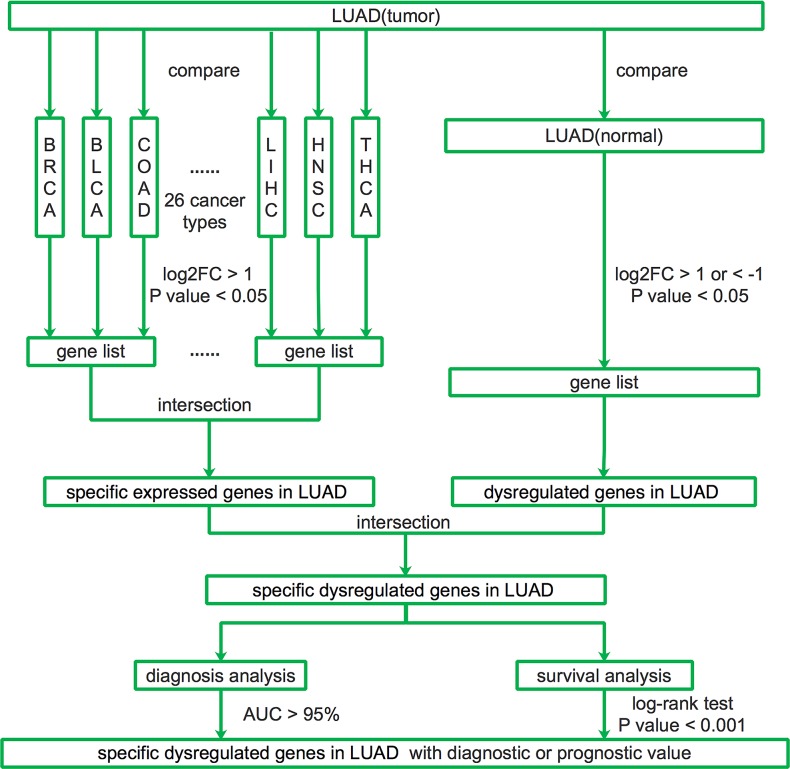
Flow chart of identifying LUAD-SDGs as diagnostic or prognostic biomarkers across RNA-seq data of 27 cancer types from TCGA

**Figure 2:**
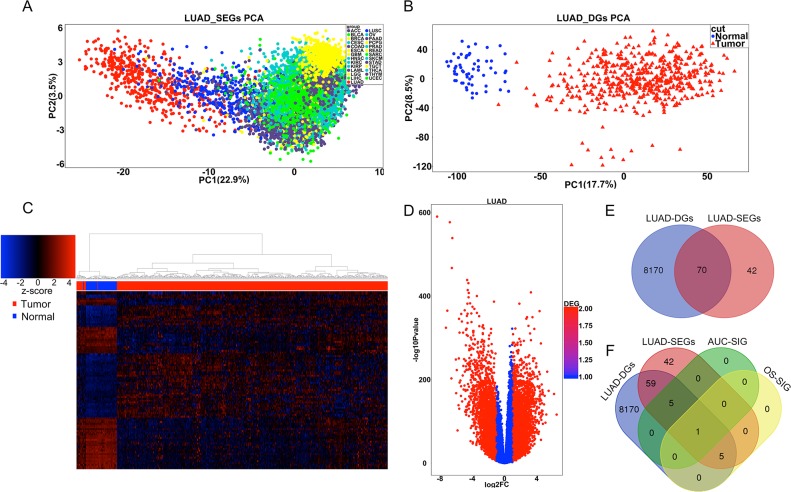
Identification of LUAD-SDGs with diagnostic or prognostic power **(A)** PCA of the full datasets for all tumor samples from 27 cancer types. LUAD samples were marked by red dots in the PCA. **(B)** PCA of the datasets for tumor and normal samples from LUAD. The tumor samples were marked by red triangles and the normal samples were marked by blue dots. **(C)** Heatmap of differentially expressed genes between tumor and normal of LUAD. The top 100 of the most differentially expressed genes were presented. **(D)** Volcano plot of the differentially expressed gene analysis. X-axis represented log2 fold change; Y-axis meant −1 × log10 of p-value for each gene. Genes with fold change >1 or <-1 and p-value < 0.05 were considered to be differentially expressed gene between tumor and normal tissues. **(E)** Venn diagram showing number of genes from differentially expressed genes (LUAD-DGs) and specifically expressed genes in LUAD (LUAD-SEGs). **(F)** Venn diagram showing the number of genes with strong diagnostic power or prognostic power among the differentially expressed genes (LUAD-DGs) and specifically expressed genes in LUAD (LUAD-SEGs). AUC-SIG(significant genes in AUC) and OS-SIG (significant genes in over survival) represented the specifically and differentially expressed genes in LUAD (LUAD-SDGs) with diagnostic values(AUC> 95%) and prognostic values respectively(P < 0.001).

To describe our study more clearly, we made a flow chart (Figure 1). We first identified the LUAD-SDGs. We found there were 112 specific expressed genes of LUAD compared with other 26 different types of cancer. The principal components analysis (PCA) demonstrated separation between LUAD and other 26 cancer types (Figure 2A). There were 8240 dysregulated expressed genes between LUAD tissues and normal tissues. PCA and hierarchical clustering showed clear separation and consistency in the expression profiles of the tumor and normal tissues of LUAD (Figure 2B, 2C). The dysregulated expressed genes of LUAD were shown with volcano plot (Figure 2D). We combined the results and demonstrated that there were 70 genes specifically dysregulated in LUAD (Figure 2E).

### Diagnostic value of specific dysregulated genes of lung adenocarcinoma (LUAD-SDGs)

**Figure 3:**
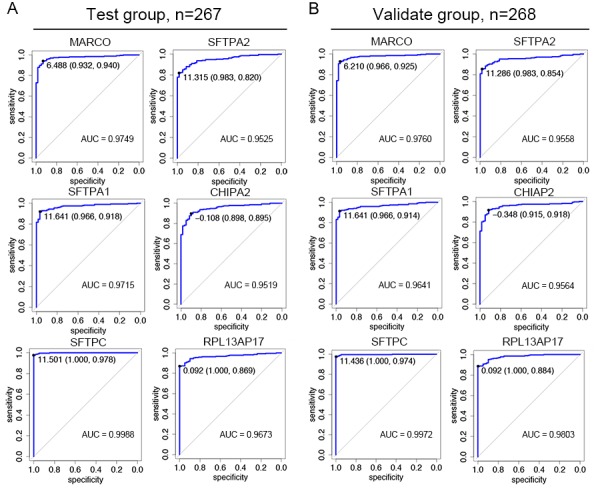
Receiver operating characteristic (ROC) plot for diagnostic value of six LUAD-SDGs **(A)** The sensitivity and specificity of MARCO, SFTPA2, SFTPA1, CHIPA2, SFTPC, and RPL13AP17 were 0.9749, 0.9525, 0.9715, 0.9519, 0.9988 and 0.9673 in the test group (n=267). **(B)** The sensitivity and specificity of MARCO, SFTPA2, SFTPA1, CHIPA2, SFTPC, and RPL13AP17 were 0.9760, 0.9558, 0.9641, 0.9564, 0.9972 and 0.9803 in the validate group (n=268).

**Figure 4:**
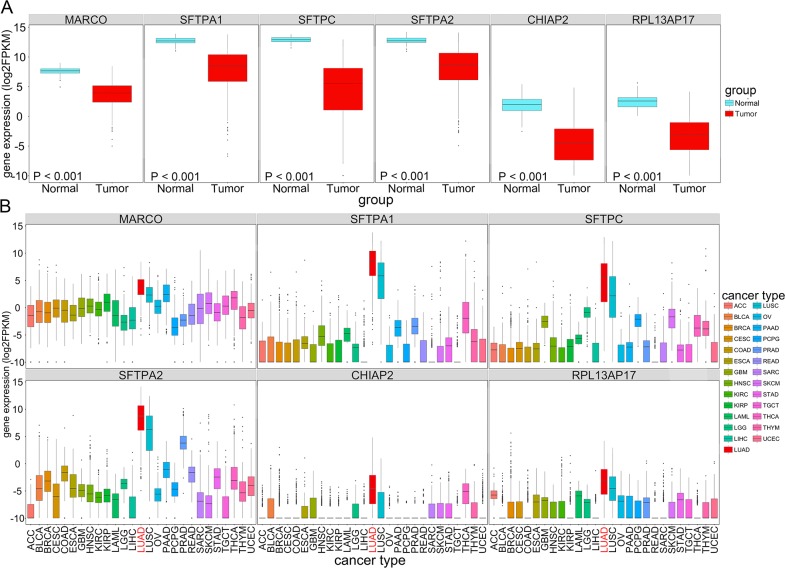
The expression (log2FPKM) of LUAD-SDGs(MARCO, SFTPA2, SFTPA1, CHIPA2, SFTPC, and RPL13AP17) with high diagnostic power in LUAD across 27 types of cancer **(A)** Box plot showing the expression of the six LUAD-SDGs in tumor were lower than that in normal of LUAD (P < 0.001). **(B)** The expression (log2FPKM) distribution of the six LUAD-SDGs in 27 cancer types and the red box represented LUAD. Compared with other tumors in 26 cancer types, the expression level of the six LUAD-SDGs in LUAD was the highest (log2FC > 1, P < 0.001).

Next we analyzed the possible diagnostic power of LUAD-SDGs in LUAD and tried to find the LUAD-SDGs with high diagnostic value. To evaluate the prediction performance of LUAD-SDGs, we performed the receiver operative curves (ROC). LUAD-SDGs with an AUC of more than 0.95 were selected as genes that may serve as a biomarker in the diagnosis of LUAD (Figure 2F). As shown in Figure 3A, we demonstrated that there were great differences between LUAD patients and control groups. The sensitivity and specificity of MARCO, SFTPA2, SFPTA1, CHIPA2, SFTPC and RPL13AP17 were 0.9749, 0.9525, 0.9715, 0.9519, 0.9988 and 0.9673 respectively in the test group. We also showed the similar results in the validate group, whose sensitivity and specificity of MARCO, SFTPA2, SFPTA1, CHIPA2, SFTPC and RPL13AP17 were 0.9760, 0.9558, 0.9641, 0.9564, 0.9972 and 0.9803 respectively (Figure 3B; Table 1). We found that SFTPC ranked top in terms of AUC in both test and validate group with average sensitivity of 100% and 97.6% specificity. These six LUAD-SDGs showed high diagnostic power in predicting LUAD, which indicated they may serve as important biomarkers in identification of LUAD. We validated the expression of the six LUAD-SDGs in the same TCGA datasets using t-test. We found that the expression of the six LUAD-SDGs was higher in LUAD than that of other tumors in 26 cancer types (log2FC > 1, P < 0.001). Compared with the expression of normal lung tissues, the expression of the six LUAD-SDGs was lower (log2FC < 1, P < 0.001) (Figure 4A, 4B), which confirmed that these genes were specific dysregulated genes in LUAD and were able to serve as diagnostic biomarkers for the diagnosis of LUAD.

**Table 1:** The detailed information of 11 LUAD-SDGs with diagnostic or prognostic values

Ensemble ID	Gene symbol	Chromosomal position	Gene type
ENSG00000019169	MARCO	Chr2: 118,942,166-118,994,660	Protein coding
ENSG00000122852	SFTPA1	Chr10: 79,610,939-79,615,455	Protein coding
ENSG00000168484	SFTPC	Chr8: 22,156,913-22,164,479	Protein coding
ENSG00000185303	SFTPA2	Chr10: 79,555,852-79,560,397	Protein coding
ENSG00000203878	CHIAP2	Chr1: 111,280,059-111,286,116	pseudogene
ENSG00000231322	RPL13AP17	Chr7: 78,347,142-78,359,458	pseudogene
ENSG00000260695	RP11-513N24.1	Chr16: 65,861,112-65,863,784	lincRNA
ENSG00000248608	RP11-206P5.2	Chr4: 25,504,997-25,506,675	pseudogene
ENSG00000168878	SFTPB	Chr2: 85,657,314-85,668,741	Protein coding
ENSG00000112175	BMP5	Chr6: 55,753,645-55,875,564	Protein coding
ENSG00000166961	MS4A15	Chr11: 60,756,953-60,776,732	Protein coding

### Prognostic role of specific dysregulated genes of lung adenocarcinoma (LUAD-SDGs)

**Figure 5:**
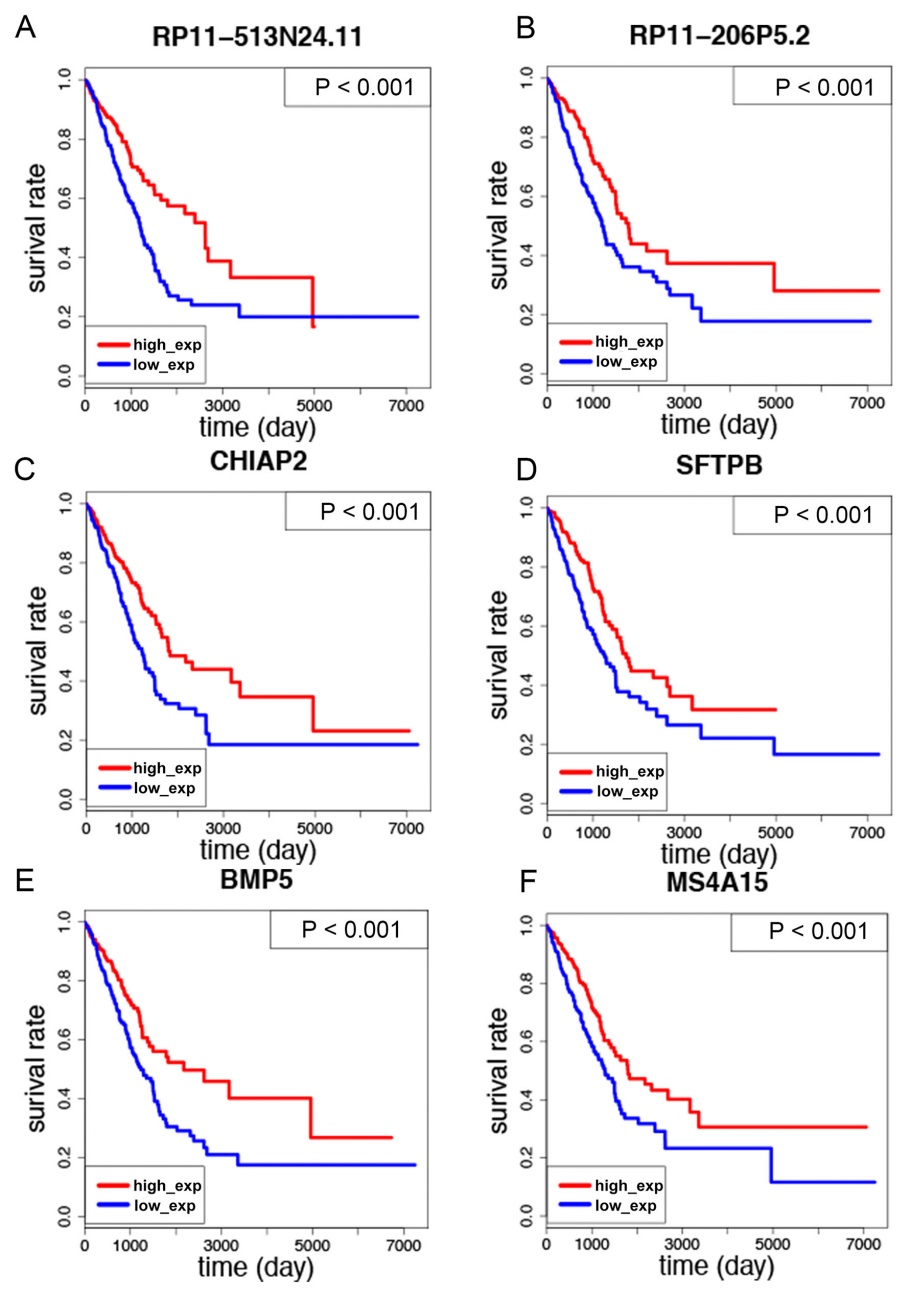
Kaplan–Meier survival curves analysis of the six LUAD-SDGs for overall survival of LUAD patients **(A-F)** The expression of six LUAD-SDGs including RP11-513N24.1, CHIAP2, RP11-206P5.2, BMP5, SFTPB and MS4A15 was positively associated with overall survival of LUAD patients (P < 0.001).

**Figure 6:**
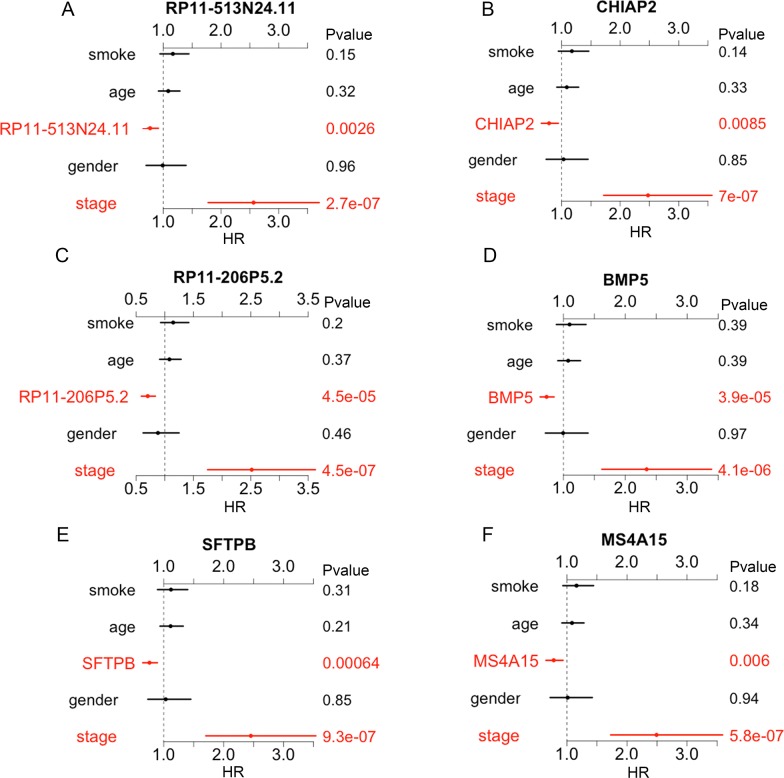
Multivariate analysis of the six OS-related LUAD-SDGs **(A-F)** The expression of the six LUAD-SDGs including RP11-513N24.1, CHIAP2, RP11-206P5.2, BMP5, SFTPB and MS4A15 served as independent predict factors in LUAD.

**Figure 7:**
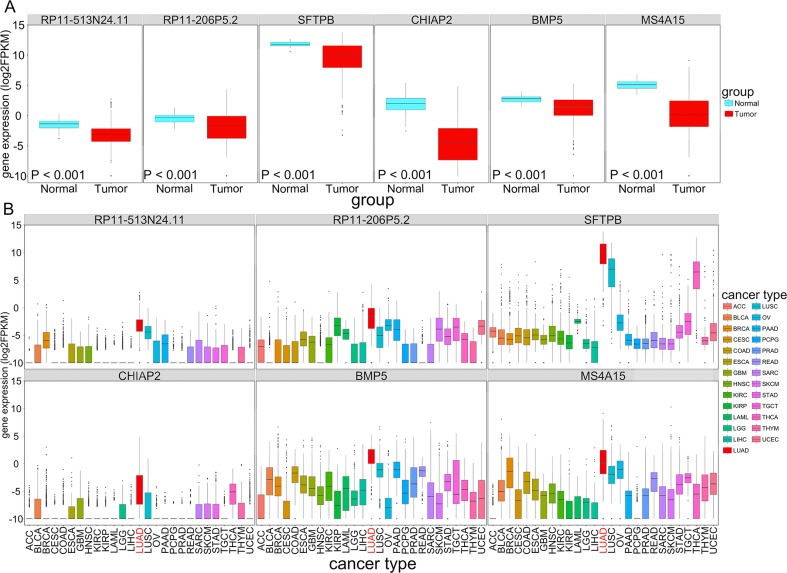
The expression (log2FPKM) of six OS-related LUAD-SDGs(RP11-513N24. 1, CHIAP2, RP11-206P5.2, BMP5, SFTPB and MS4A15) across 27 types of cancer **(A)** Box plot showed the expression of the six LUAD-SDGs in tumor were lower than that in normal of LUAD. **(B)** The expression (log2FPKM) distribution of the six genes in 27 cancer types and the red box represented LUAD. Compared with other tumors in 26 cancer types, the expression level of the six LUAD-SDGs in LUAD was the highest (log2FC > 1, P < 0.001).

Then we sought to investigate the correlation between all of the 70 LUAD-SDGs and overall survival of LUAD patients. We identified that there were six LUAD-SDGs whose expression were associated with the overall survival of LUAD patients, including RP11-513N24.11, RP11-206P5.2, SFTPB, CHIAP2, BMP5 and MS4A15 (Table 1). The Kaplan–Meier curves demonstrated that LUAD patients with higher expression of the six LUAD-SDGs had better survivals than those with low expression of the six LUAD-SDGs (log-Rank test, P < 0.001) (Figure 5A–5F). In the univariate Cox regression analysis, we found that the six LUAD-SDGs were significantly associated with overall survival. Then we performed the multivariate Cox regression analysis to identify the independent prognostic role of the six LUAD-SDGs by adjusting other significant covariates including gender, stage, smoke and age. The results showed that the six LUAD-SDGs were independent prognostic factors in overall survival and were protective genes (HR < 1) for LUAD (Figure 6A–6F; Table 2). Compared with other 26 types of cancer, the expression level of the six LUAD-SDGs in LUAD was the highest (log2FC > 1, P < 0.001). In non-cancer tissue of LUAD, the six LUAD-SDGs had higher expression level than the cancer tissue of LUAD. All the results suggested that the six LUAD-SDGs may act as special prognostic biomarkers for LUAD patients. (Figure 7A, 7B).

**Table 2:** Univariate and multivariate Cox regression analysis of the six OS-related LUAD-SDGs and the six-gene signature

Variables	Univariate analysis			Multivariate analysis		
	HR	95% CI of HR	P value	HR	95% CI of HR	P value
**RP11-513N24.1**	0.7601	0.6433—0.8982	0.0013	0.7733	0.6543—0.9140	0.0026
gender	1.0508	0.7569—1.4589	0.7672	0.991154	0.7089—1.3859	0.9586
stage	2.5857	1.8110—3.6919	1.71E-07	2.563875	1.7908—3.6707	2.72E-07
smoke	1.1947	0.9755—1.4631	0.0854	1.167386	0.9473—1.4385	0.1464
age	1.0686	0.9020—1.2660	0.4431	1.087631	0.9209—1.2845	0.3224
**RP11-206P5.2**	0.6963	0.5915—0.8198	1.38E-05	0.7008	0.5908—0.8313	4.47E-05
gender	1.0508	0.7569—1.4589	0.7672	0.8792	0.6243—1.2382	0.4612
stage	2.5857	1.8110—3.6919	1.71E-07	2.5147	1.7578—3.5974	4.48E-07
smoke	1.1947	0.9755—1.4631	0.0854	1.1448	0.9291—1.4104	0.2042
age	1.0686	0.9020—1.2660	0.4431	1.0796	0.9122—1.2778	0.3731
**SFTPB**	0.737	0.6346—0.8559	0.0001	0.7627	0.6529—0.8909	0.0006
gender	1.0508	0.7569—1.4589	0.7672	1.0336	0.7403—1.4432	0.8459
stage	2.5857	1.8110—3.6919	1.71E-07	2.4546	1.7147—3.5138	9.29E-07
smoke	1.1947	0.9755—1.4631	0.0854	1.1212	0.9008—1.3955	0.3058
age	1.0686	0.9020—1.2660	0.4431	1.1153	0.9412—1.3215	0.2077
**CHIAP2**	0.7585	0.6339—0.9075	0.0025	0.7845	0.6548—0.9399	0.0085
gender	1.0508	0.7569—1.4589	0.7672	1.0321	0.7392—1.4411	0.8529
stage	2.5857	1.8110—3.6919	1.71E-07	2.4762	1.7307—3.5427	6.99E-07
smoke	1.1947	0.9755—1.4631	0.0854	1.1744	0.9464—1.4572	0.1444
age	1.0686	0.9020—1.2660	0.4431	1.0878	0.9199—1.2863	0.3251
**BMP5**	0.6858	0.5925—0.7938	4.25E-07	0.7268	0.6243—0.8461	3.9E-05
gender	1.0508	0.7569—1.4589	0.7672	0.9943	0.7121—1.3883	0.9732
stage	2.5857	1.8110—3.6919	1.71E-07	2.3439	1.6316—3.3672	4.1E-06
smoke	1.1947	0.9755—1.4631	0.0854	1.0986	0.8883—1.3588	0.3857
age	1.0686	0.9020—1.2660	0.4431	1.0760	0.9110—1.2710	0.3882
**MS4A15**	0.7568	0.636—0.9007	0.0017	0.7801	0.6536—0.9312	0.006
gender	1.0508	0.7569—1.4589	0.7672	1.0126	0.7255—1.4133	0.9414
stage	2.5857	1.8110—3.6919	1.71E-07	2.4917	1.7418—3.5646	5.81E-07
smoke	1.1947	0.9755—1.4631	0.0854	1.1598	0.9359—1.4373	0.1755
age	1.0686	0.9020—1.2660	0.4431	1.0846	0.9190—1.2800	0.3367
**Risk score**	1.5815	1.3658—1.8313	9.02E-10	1.5129	1.2988—1.7624	1.05E-07
gender	1.0508	0.7569—1.4589	0.7672	0.9217	0.6595—1.2883	0.6333
stage	2.5857	1.8110—3.6919	1.71E-07	2.2685	1.5805—3.2558	8.87E-06
smoke	1.1947	0.9755—1.4631	0.0854	1.0906	0.8785—1.3539	0.4317
age	1.0686	0.9020—1.2660	0.4431	1.0922	0.9249—1.2898	0.2983

### The predictive role of a six-gene signature in LUAD

**Figure 8:**
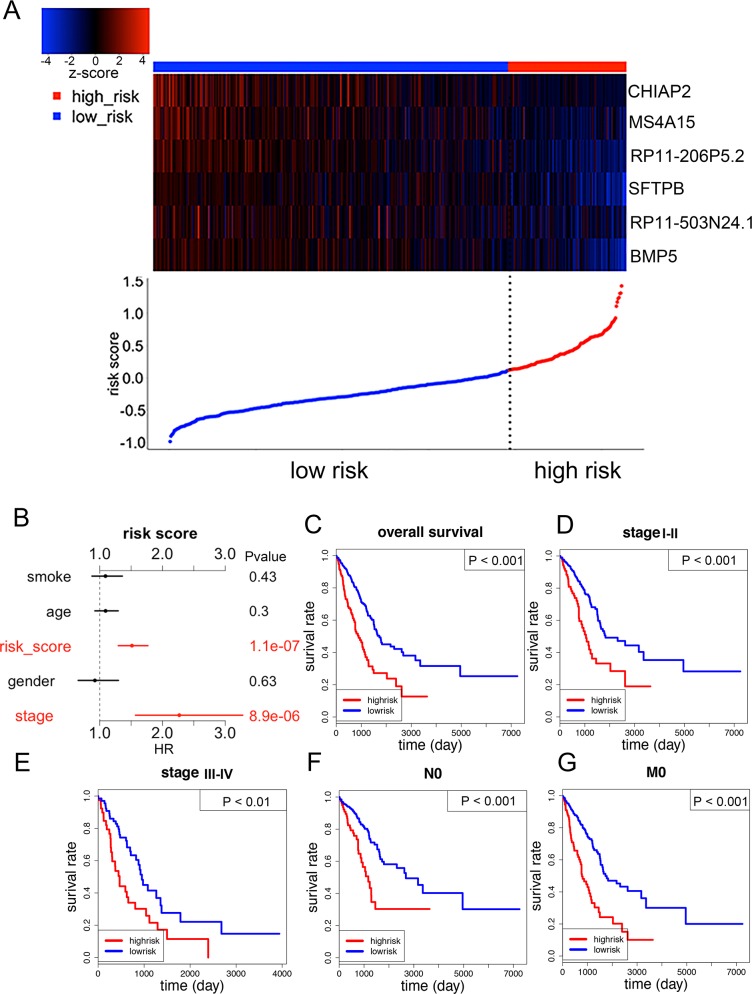
The six-gene signature predicted overall survival of patients with LUAD **(A)** The six genes’ expression and risk score distribution, with red indicating higher expression and blue indicating lower expression. The risk scores for all patients in LUAD were plotted in ascending order and marked as low risk (blue) or high risk (red). **(B)** Multivariate Cox regression analysis showing the risk score of the six-gene signature can serve as an independent prognostic factor in LUAD. **(C)** The Kaplan-Meier curves for all LUAD patients stratified by six-gene signature in high and low risk. **(D-G)** Kaplan-Meier curves of overall survival in the stage I-II, stage III-IV, N0 and M0 cohort stratified by six-gene signature in high and low risk.

We used the six OS-related LUAD-SDGs to construct a six-gene signature. A risk score was created based on a linear combination of the expression profiles of prognostic genes weighted by estimated regression coefficient, a risk score was created as follows: Risk score = (−0.013^*^expression level of RP11-513N24.11) + (−0.066^*^expression level of RP11-206P5.2) + (−0.029^*^expression level of SFTPB) + (−0.028^*^expression level of CHIAP2) + (−0.087^*^expression level of BMP5) + (−0.029^*^expression level of MS4A15). Then, patients were divided into high-risk group (n = 131) and low-risk group (n = 392) using the 75^th^ percentile risk score as the cutoff point to investigate the prognostic role of the six-gene signature in overall survival. We observed that patients in the high-risk group had lower expression of the six genes compared with low-risk group (Figure 8A). This was consistent with the results of multivariate Cox regression analysis of the six genes, which demonstrated that all of them were protective genes (Figure 6A–6F). The univariate analysis showed that the six-gene risk score was significantly related to prognosis of LUAD patients (Table 2). The multivariate analysis indicated that the risk score statistically significantly stratified the patients for overall survival (HR =1.5129, 95%CI = 1.2988-1.7624, P < 0.0001), independent of age, gender, smoke and pathological stage. (Figure 8B, 8C; Table 2). We also evaluated the six-gene prognostic signature in patients at pathological stage I-II, pathological stage IV-III, N0 (without lymph node metastasis), M0(without distant metastasis). As a result, the stratification analysis showed that the six-gene signature could predict patients with different prognosis in different subgroups including pathological stage I-II, pathological stage IV-III, N0, M0 (Figure 8D–8G).

## DISCUSSION

In our study, we first compared the expression of genes in 27 cancer types including LUAD between tumor and normal. As a result, all 70 LUAD-SDGs were identified. As we know, there were several known cancer-specific biomarkers that had already been translated into clinical diagnostic and prognostic signatures. For example, compared with other malignancies, alpha-fetoprotein (AFP) was specific expressed in hepatocellular carcinoma [14]. Besides, FUMIO *et al* showed hepatocellular carcinoma patients with normal AFP values had a better survival than those with high values, which indicated that AFP was a useful prognostic signature for hepatocellular carcinoma patients [15]. Welch *et al* showed prostate specific antigen (PSA) screening increased diagnosis of prostate cancer in patients younger than 50 years in the USA from 1986 to 2005 [16]. Inspired by these cancer-specific biomarkers, we are interested in whether the LUAD-SDGs can serve as good biomarkers for patients’ diagnosis and prognosis.

In order to explore the above questions, we conducted the ROC to evaluate the diagnostic role of LUAD-SDGs. The cutoff point was selected (AUC > 95%) and only six genes: MARCO, SFTPA1, SFTPA2, CHIPA2, SFTPC, and RPL13AP17 reached this condition. Among the six LUAD-SDGs, surfactant protein A including SFTPA1 and SFTPA2 were reported closely associated with lung cancer. It has been reported that SFTPA1 mutation was associated with familial idiopathic interstitial pneumonia and lung cancer, which shed light on the key role of SFTPA1 in the pathogenesis of several chronic respiratory diseases, especially in lung cancer [17]. Genetic defects in SFTPA2 were demonstrated closely related to lung cancer [18]. Surfactant protein A plays an important role in the maintenance of normal lung function, and the abnormal surfactant protein A may lead to the disorder of lung innate immunity and cause the occurrence of lung cancer. What's more, SFTPA1, SFTPA2 and SFTPC, surfactant genes which have been reported to express exclusively in type II penumocytes, have showed their unique roles in LUAD [19]. Meanwhile, there are some reports approving that surfactant genes such as pro–surfactant protein B (pro-SFTPB) was a promising blood biomarker for non-small-cell lung cancer. *Don D. Sin* et al showed plasma levels of pro-SFTPB were associated with early-stage lung cancer, indicating it could be used in predicting early-stage NSCLC patients. Besides, *William R. Wikoff* et al found combination of pro-SFTPB and N1, N12-diacetylspermine had a high sensitivity and specificity in diagnosing NSCLC [20, 21]. It is a great inspiration to us, as some of the six LUAD-SDGs may have the potential to become the diagnostic biomarkers for LUAD or to enhance the accuracy when combined with pathological diagnosis.

Then we explored the possible roles of LUAD-SDGs in LUAD patients’ prognosis. Among the 70 LUAD-SDGs, six of them were identified significantly associated with patients’ prognosis. In some studies, the expression of BMP5 demonstrated significantly lower in LUAD tissues than that in normal lung tissues, which was consistent with our result [22]. While there was no report about the prognostic function of BMP5, Pro–surfactant protein B (pro-SFTPB) was reported as a promising blood biomarker for non-small-cell lung cancer [20, 21]. Ayumu *et al* demonstrated that increasing concentration of plasma pro-SFTPB was associated with higher lung cancer risk, which confirmed that SFTPB might be a valuable biomarker in lung cancer risk prediction models [20, 23]. Besides, CHIAP2 was also reported associated with LUAD patients’ prognosis. JING SUI et al identified that CHIAP2 was a cancer specific lncRNA and was positively correlated with OS of LUAD [24]. However, except for CHIAP2 and SFTPB, other four LUAD-SDGs did not have any report about the relationship between their expressions and LUAD patients’ prognosis. Our study explored that the six LUAD-SDGs: BMP5, SFTPB, CHIAP2, RP11-513N24.1, RP11-206P5.2 and MS4A15 which may be positively associated with survival. In order to explore whether the six LUAD-SDGs can serve as independent prognostic factors for overall survival in LUAD, the multivariate Cox regression analysis was performed. In conclusion, the results showed that the six LUAD-SDGs were protective genes (HR < 1) for LUAD and played independent prognostic roles in LUAD.

Finally, we used the six survival-related LUAD-SDGs to construct a six-gene signature, which proved to be a prognostic biomarker for LUAD. We also performed Cox regression analyses to evaluate whether the prognostic power of the six-gene signature was independent of other clinical variables, such as tumor stage, smoking, age and gender. We demonstrated that the prognostic power of the six-gene signature was independent after taking other clinical variables into account. Besides, in the stratified analyses, patients were classified into high-risk and low-risk groups. Similar prognostic power was tested in the early patients (stage I-II), the advanced stage(III-IV), N0 (without lymph metastasis), M0 (without distant metastasis). In general, all the results suggested that the six-gene signature owned a good independent prognostic power even when other clinical variables are taken into account. Sudhanshu Shukla *et al* first presented the RNA-seq prognostic signature consisting of four genes for LUAD [25]. However, in our work, we first demonstrated that LUAD-SDGs could also act as good prognostic signatures for LUAD, which could provide insights into new prognostic biomarkers exploration.

In conclusion, by analyzing the large scale RNA-seq of 10098 tumor tissues across 27 TCGA cancer types, we identified six LUAD-SDGs which showed diagnosis power. Meanwhile, other six LUAD-SDGs were significantly associated with LUAD patients’ prognosis. Moreover, the six-gene signature could effectively predict patients’ prognosis and function as a new independent prognostic biomarker for LUAD. But we have to admit that there are limitations in our study. Firstly, the main limitation of the study is the lack of cross-validation cohort from other databases. Validating these genes in a larger cohort of LUAD patients can make the LUAD-SDGs for diagnosis and prognosis more convincing. Secondly, the six LUAD-SDGs with diagnosis power should be validated in serums samples to own their true diagnosis value. But these limitations are certain to be solved in future investigation. [26–39]

## MATERIALS AND METHODS

### RNA-seq data collection and processing

Transcriptome data and clinical data were downloaded from The Cancer Genome Atlas (TCGA) data portal (https://cancergenome.nih.gov/). We chose 27 cancer types, all of which included over 100 tumor samples. We collected and analyzed data from 10098 tumor samples, which contained 535 tumor samples with related normal samples and clinical information in this study (Table 3). The transcriptomic correlation of RNA expression was determined by RNA-seq (Fragments Per Kilobase Million [FPKM]) and the expression profiles were normalized by log2 transformed. To ensure the reliability of the detection, we removed genes whose FPKM values over 50% of the samples is equal to 0.

**Table 3:** List of the 27 cancer types analyzed

Cancer types	Abbreviation	RNA-seq		Reference
		No. of normal	No. of tumor	
Adrenocortical Carcinoma	ACC	2	185	NA
Bladder Urothelial Carcinoma	BLCA	19	414	[26]
Breast Invasive Carcinoma	BRCA	113	1109	[27]
Cervical Squamous Cell Carcinoma and Endocervical Adenocarcinoma	CESC	3	306	NA
Colon Adenocarcinoma	COAD	41	480	[28]
Esophageal Carcinoma	ESCA	11	162	NA
Glioblastoma Multiforme	GBM	5	169	[29]
Head and Neck Squamous Cell Carcinoma	HNSC	44	502	[30]
Kidney Renal Clear Cell Carcinoma	KIRC	72	539	[31]
Kidney Renal Papillary Cell Carcinoma	KIRP	32	289	NA
Acute Myeloid Leukemia	LAML	0	151	[32]
Brain Lower Grade Glioma	LGG	0	529	NA
Liver Hepatocellular Carcinoma	LIHC	50	374	NA
Lung Adenocarcinoma	LUAD	59	535	[33]
Lung Squamous Cell Carcinoma	LUSC	49	502	[34]
Ovarian Serous Cystadenocarcinoma	OV	0	379	[35]
Pancreatic Adenocarcinoma	PAAD	4	178	NA
Pheochromocytoma and Paraganglioma	PCPG	3	183	NA
Prostate Adenocarcinoma	PRAD	52	499	[36]
Rectum Adenocarcinoma	READ	10	167	[28]
Sarcoma	SARC	2	263	NA
Skin Cutaneous Melanoma	SKCM	1	471	[37]
Stomach Adenocarcinoma	STAD	32	375	[38]
Testicular Germ Cell Tumors	TGCT	0	156	NA
Thyroid Carcinoma	THCA	58	510	NA
Thymoma	THYM	2	119	NA
Uterine Corpus Endometrial Carcinoma	UCEC	35	552	[39]
**Total**		**699**	**10098**	

### Analysis of specific dysregulated genes in LUAD

Firstly the dysregulated genes in LUAD between tumor and normal were identified using t-test (P value< 0.05, log2FC > 1 or <-1). Then the specific expressed genes of LUAD were acquired by comparing the expression of the genes between LUAD and other tumors in 26 cancer types one by one (P< 0.05, log2FC > 1). Specific dysregulated genes in LUAD were selected, which were specific expressed in LUAD among 27 cancer types and dysregulated expressed between tumor and normal of LUAD.

### Diagnosis and survival analysis

All 535 LUAD patients data were randomly divided into test group (n=267) and validate group (n=268). The receiver operator characteristic (ROC) curves were used to evaluate the specificity and sensitivity of the LUAD-SDGs. The genes with high diagnostic power were selected (AUC > 95%). Meanwhile, for overall survival analysis, Kaplan-Meier survival and log-rank test were used to compare significant differences between subgroups with univariate analysis.

### Construction of a prognostic signature

Univariate and multivariate Cox proportional hazards regression were used to assess the LUAD-SDGs whose expressions were significantly associated with patients’ survival. Hazard ratios (HRs) from multivariable cox regression analysis were used to identify protective (HR < 1) or risky genes (HR > 1). Subsequently, a prognostic signature was constructed based on a linear combination of the expression profiles of prognostic genes weighted by the estimated regression coefficient [25, 40–42].

$$\text{Risk Score}=\sum_{i=1}^{N}\left(\text{Exp}i\;*\text{ Coe}i\right)$$

N was the number of prognostic genes, Exp*i* was the expression of genes and Coe*i* was the coefficient value. A risk score was constructed with the regression coefficients from this model and 75^th^ percentile was chosen as the threshold.

### Statistical analysis

T-test was used to compare the dysregulated genes in LUAD between tumor and normal. Besides, it was also used to analyze specific expressed genes in LUAD compared with other tumors in 26 cancer types. The receiver operating characteristic (ROC) curve was performed using R package “pROC”[43]. The Kaplan–Meier method with a log- rank test was used to assess patients’ survival using the R packages “survival”[44]. The univariate and multivariable Cox proportional hazards regression were performed using the R packages “BhGLM”[45]. Heatmaps were generated with z-score normalization with each column using R packages “gplots”[46]. All analyses were performed using R software (version 3.2.2). A statistically significant difference when P value < 0.05 was considered.

## References

[1] Torre LA, Bray F, Siegel RL, Ferlay J, Lortet-Tieulent J, Jemal A (2015). Global cancer statistics, 2012. CA Cancer J Clin.

[2] Meza R, Meernik C, Jeon J, Cote ML (2015). Lung cancer incidence trends by gender, race and histology in the United States, 1973-2010. PLoS One.

[3] Alberg AJ, Brock MV, Ford JG, Samet JM, Spivack SD (2013). Epidemiology of lung cancer: diagnosis and management of lung cancer: American College of Chest Physicians evidence-based clinical practice guidelines. Chest.

[4] Sun S, Schiller JH, Gazdar AF (2007). Lung cancer in never smokers--a different disease. Nat Rev Cancer.

[5] Saito M, Shiraishi K, Kunitoh H, Takenoshita S, Yokota J, Kohno T (2016). Gene aberrations for precision medicine against lung adenocarcinoma. Cancer Sci.

[6] Khoo C, Rogers TM, Fellowes A, Bell A, Fox S (2015). Molecular methods for somatic mutation testing in lung adenocarcinoma: EGFR and beyond. Transl Lung Cancer Res.

[7] Siegelin MD, Borczuk AC (2014). Epidermal growth factor receptor mutations in lung adenocarcinoma. Lab Invest.

[8] Wang Z, Gerstein M, Snyder M (2009). RNA-seq: a revolutionary tool for transcriptomics. Nat Rev Genet.

[9] Wei IH, Shi Y, Jiang H, Kumar-Sinha C, Chinnaiyan AM (2014). RNA-Seq accurately identifies cancer biomarker signatures to distinguish tissue of origin. Neoplasia.

[10] Feng A, Tu Z, Yin B (2016). The effect of HMGB1 on the clinicopathological and prognostic features of non-small cell lung cancer. Oncotarget.

[11] Gutschner T, Hammerle M, Eissmann M, Hsu J, Kim Y, Hung G, Revenko A, Arun G, Stentrup M, Gross M, Zornig M, MacLeod AR, Spector DL (2013). The noncoding RNA MALAT1 is a critical regulator of the metastasis phenotype of lung cancer cells. Cancer Res.

[12] Sun M, Liu XH, Wang KM, Nie FQ, Kong R, Yang JS, Xia R, Xu TP, Jin FY, Liu ZJ, Chen JF, Zhang EB, De W Wang ZX (2014). Downregulation of BRAF activated non-coding RNA is associated with poor prognosis for non-small cell lung cancer and promotes metastasis by affecting epithelial-mesenchymal transition. Mol Cancer.

[13] Gao F, Chang J, Wang H, Zhang G (2014). Potential diagnostic value of miR-155 in serum from lung adenocarcinoma patients. Oncol Rep.

[14] Chen DS, Sung JL (1977). Serum alphafetoprotein in hepatocellular carcinoma. Cancer.

[15] Nomura F, Ohnishi K, Tanabe Y (1989). Clinical features and prognosis of hepatocellular carcinoma with reference to serum alpha-fetoprotein levels. Analysis of 606 patients. Cancer.

[16] Welch HG, Albertsen PC (2009). Prostate cancer diagnosis and treatment after the introduction of prostate-specific antigen screening: 1986-2005. J Natl Cancer Inst.

[17] Nathan N, Giraud V, Picard C, Nunes H, Dastot-Le Moal F, Copin B, Galeron L, De Ligniville A, Kuziner N, Reynaud-Gaubert M, Valeyre D, Couderc LJ, Chinet T (2016). Germline SFTPA1 mutation in familial idiopathic interstitial pneumonia and lung cancer. Hum Mol Genet.

[18] Wang Y, Kuan PJ, Xing C, Cronkhite JT, Torres F, Rosenblatt RL, DiMaio JM, Kinch LN, Grishin NV, Garcia CK (2009). Genetic defects in surfactant protein A2 are associated with pulmonary fibrosis and lung cancer. Am J Hum Genet.

[19] McCall MN, Illei PB, Halushka MK (2016). Complex sources of variation in tissue expression data: analysis of the GTEx lung transcriptome. Am J Hum Genet.

[20] Sin DD, Tammemagi CM, Lam S, Barnett MJ, Duan X, Tam A, Auman H, Feng Z, Goodman GE, Hanash S, Taguchi A (2013). Pro-surfactant protein B as a biomarker for lung cancer prediction. J Clin Oncol.

[21] Wikoff WR, Hanash S, DeFelice B, Miyamoto S, Barnett M, Zhao Y, Goodman G, Feng Z, Gandara D, Fiehn O, Taguchi A (2015). Diacetylspermine is a novel prediagnostic serum biomarker for non-small-cell lung cancer and has additive performance with pro-surfactant protein B. J Clin Oncol.

[22] Deng T, Lin D, Zhang M, Zhao Q, Li W, Zhong B, Deng Y, Fu X (2015). Differential expression of bone morphogenetic protein 5 in human lung squamous cell carcinoma and adenocarcinoma. Acta Biochim Biophys Sin (Shanghai).

[23] Taguchi A, Hanash S, Rundle A, McKeague IW, Tang D, Darakjy S, Gaziano JM, Sesso HD, Perera F (2013). Circulating pro-surfactant protein B as a risk biomarker for lung cancer. Cancer Epidemiol Biomarkers Prev.

[24] Sui J, Li YH, Zhang YQ, Li CY, Shen X, Yao WZ, Peng H, Hong WW, Yin LH, Pu YP, Liang GY (2016). Integrated analysis of long non-coding RNAassociated ceRNA network reveals potential lncRNA biomarkers in human lung adenocarcinoma. Int J Oncol.

[25] Shukla S, Evans JR, Malik R, Feng FY, Dhanasekaran SM, Cao X, Chen G, Beer DG, Jiang H, Chinnaiyan AM (2016). Development of a RNA-Seq based prognostic signature in lung adenocarcinoma. J Natl Cancer Inst.

[26] Cancer Genome Atlas Research Network (2014). Comprehensive molecular characterization of urothelial bladder carcinoma. Nature.

[27] Cancer Genome Atlas Network (2012). Comprehensive molecular portraits of human breast tumours. Nature.

[28] Cancer Genome Atlas Network (2012). Comprehensive molecular characterization of human colon and rectal cancer. Nature.

[29] Cancer Genome Atlas Research Network (2008). Comprehensive genomic characterization defines human glioblastoma genes and core pathways. Nature.

[30] Cancer Genome Atlas Network (2015). Comprehensive genomic characterization of head and neck squamous cell carcinomas. Nature.

[31] Cancer Genome Atlas Research Network (2013). Comprehensive molecular characterization of clear cell renal cell carcinoma. Nature.

[32] Cancer Genome Atlas Research Network (2013). Genomic and epigenomic landscapes of adult de novo acute myeloid leukemia. N Engl J Med.

[33] Cancer Genome Atlas Research Network (2014). Comprehensive molecular profiling of lung adenocarcinoma. Nature.

[34] Cancer Genome Atlas Research Network (2012). Comprehensive genomic characterization of squamous cell lung cancers. Nature.

[35] Cancer Genome Atlas Research Network (2011). Integrated genomic analyses of ovarian carcinoma. Nature.

[36] Cancer Genome Atlas Research Network (2015). The molecular taxonomy of primary prostate cancer. Cell.

[37] Cancer Genome Atlas Network (2015). Genomic classification of cutaneous melanoma. Cell.

[38] Cancer Genome Atlas Research Network (2014). Comprehensive molecular characterization of gastric adenocarcinoma. Nature.

[39] Kandoth C, Schultz N, Cherniack AD, Akbani R, Liu Y, Shen H, Robertson AG, Pashtan I, Shen R, Benz CC, Yau C, Laird PW, Ding L, Cancer Genome Atlas Research Network (2013). Integrated genomic characterization of endometrial carcinoma. Nature.

[40] Lossos IS, Czerwinski DK, Alizadeh AA, Wechser MA, Tibshirani R, Botstein D, Levy R (2004). Prediction of survival in diffuse large-B-cell lymphoma based on the expression of six genes. N Engl J Med.

[41] Chen HY, Yu SL, Chen CH, Chang GC, Chen CY, Yuan A, Cheng CL, Wang CH, Terng HJ, Kao SF, Chan WK, Li HN, Liu CC (2007). A five-gene signature and clinical outcome in non-small-cell lung cancer. N Engl J Med.

[42] Beer DG, Kardia SL, Huang CC, Giordano TJ, Levin AM, Misek DE, Lin L, Chen G, Gharib TG, Thomas DG, Lizyness ML, Kuick R, Hayasaka S (2002). Gene-expression profiles predict survival of patients with lung adenocarcinoma. Nat Med.

[43] Robin X, Turck N, Hainard A, Tiberti N, Lisacek F, Sanchez JC, Müller M, Siegert S Package “pROC.”. http://www.r-project.org.

[44] Therneau MT, Thomas LA Survival analysis. http://www.r-project.org.

[45] Yi N Package “BhGLM.”. http://www.ssg.uab.edu/bhglm/.

[46] Warnes GR, Bolker B, Bonebakker L, Gentleman R, Liaw WH, Lumley T, Maechler M, Magnusson A, Moeller S, Schwartz M, Venables B Packages “gplots.”. http://www.r-project.org.

